# Incidence of All-Cause and Cardiovascular Mortality Predicted by Symmetric Dimethylarginine in the Population-Based Study of Health in Pomerania

**DOI:** 10.1371/journal.pone.0096875

**Published:** 2014-05-12

**Authors:** Edzard Schwedhelm, Henri Wallaschofski, Dorothee Atzler, Marcus Dörr, Matthias Nauck, Uwe Völker, Heyo K. Kroemer, Henry Völzke, Rainer H. Böger, Nele Friedrich

**Affiliations:** 1 Institute of Clinical Pharmacology and Toxicology, University Medical Center Hamburg-Eppendorf, Hamburg, Germany; 2 Cardiovascular Research Center, University Medical Center Hamburg-Eppendorf, Hamburg, Germany; 3 DZHK (German Centre for Cardiovascular Research), Partner Site Hamburg/Lübeck/Kiel, Hamburg, Germany; 4 Institute of Clinical Chemistry and Laboratory Medicine, University Medicine Greifswald, Greifswald, Germany; 5 DZHK (German Centre for Cardiovascular Research), Partner Site, Greifswald, Germany; 6 Department of Internal Medicine B/Cardiology, University Medicine Greifswald, Greifswald, Germany; 7 Interfaculty Institute for Genetics and Functional Genomics, University Medicine Greifswald, Greifswald, Germany; 8 DZHK (German Centre for Cardiovascular Research), Partner Site Göttingen, Göttingen, Germany; 9 Institute of Community Medicine, Department SHIP/Clinical Epidemiology, University Medicine Greifswald, Greifswald, Germany; Kagoshima University Graduate School of Medical and Dental Sciences, Japan

## Abstract

**Background:**

L-Arginine and its dimethylated derivatives asymmetric dimethylarginine (ADMA) and symmetric dimethylarginine (SDMA) have been associated with cardiovascular (CV) and all-cause mortality in populations at risk. The present study aimed to investigate the prognostic value of L-arginine and its derivatives in the general population.

**Methods and Results:**

We evaluated 3,952 individuals (1,936 men and 2,016 women) aged 20–81 (median (IQR) 51 (37; 64) years) from the population-based Study of Health in Pomerania (SHIP). Associations of continuous [per standard deviation (SD) increase] and categorized (age- and sex-specific tertiles) serum L-arginine, ADMA, and SDMA concentrations with all-cause and cause-specific mortality were analysed. During a median (IQR) follow-up period of 10.1 (9.3; 10.8) years (38,476 person-years), 426 deaths (10.8%) were observed, including 139 CV deaths (3.5%), and 150 cancer deaths (3.8%). After multivariable adjustment, we revealed a positive association of SDMA with all-cause [hazard ratio (HR) per SD increase: 1.16, 95% confidence interval (CI): 1.07–1.25] and CV mortality [HR: 1.19, 95% CI: 1.05–1.35]. In contrast, we did not observe any association of SDMA with cancer mortality. Neither L-arginine nor ADMA were associated with all-cause or CV mortality.

**Conclusion:**

SDMA, but not ADMA, is an independent predictor of all-cause and CV mortality in a large population-based cohort of European ancestry.

## Introduction

L-Arginine is the precursor of the vasodilator nitric oxide (NO), which has been shown to have anti-aggregatory, anti-inflammatory, and anti-atherosclerotic effects [1]. L-Arginine and its methylated derivatives asymmetric dimethylarginine (ADMA) and symmetric dimethylarginine (SDMA) are constituents of human nutrition, with L-arginine as a proteinogenic amino acid being ubiquitous present whilst conspicuous amounts of ADMA and SDMA were found in some vegetables like soy beans [2]. The beneficial effect of L-arginine supplementation for cardiovascular (CV) health is controversial, and oral administration results in only modest increases of L-arginine blood concentrations due to considerable pre-systemic elimination [3], [4].

Even though L-arginine is the main precursor of NO, regulation of NO production is not limited by circulating L-arginine,but by intracellular substrate and co-factor availability, NO synthase (NOS) activation by phosphorylation, and NOS inhibition by e.g., the endogenously methylated L-arginine derivate ADMA, but not SDMA [5]. Similar to ADMA, SDMA inhibits cationic amino acid transport into cells leading to limited cellular L-arginine uptake [6], [7]. Besides the influence on NO regulation, ADMA and SDMA have been demonstrated to be markers of CV outcome and mortality in several studies among patients with intermediate and high CV risk (reviewed in [8]). Data of the Framingham Heart Study indicates that ADMA predicts all-cause mortality in a community-based cohort [9]. SDMA was identified as an independent predictor of all-cause and CV mortality in the multiethnic population-based cohort of the Dallas Heart Study [10]. However, other prospective data from epidemiologic cohorts is sparse. Therefore the aim of the present study was to investigate the association of ADMA and SDMA with all-cause and CV mortality in the population-based Study of Health in Pomerania (SHIP).

## Methods

### Design and subjects

SHIP is a longitudinal population-based cohort study in West Pomerania, a region in Northeast Germany [11], [12]. The total population of West Pomerania selected for SHIP comprised 212,157 inhabitants. The sampling of the SHIP cohort was performed from population registries, where all German citizens are registered. A two-stage cluster sampling method adopted from the WHO MONICA Project Augsburg, Germany yielded 12 five-year age strata (20 to 79 years) for both genders, each including 292 individuals [13]. Data collection started in October 1997 and was finished in March 2001. The net sample (after exclusion of migrated or deceased persons) comprised 6,267 eligible subjects. Finally, 2,116 men and 2,192 women (response proportion 69%) participated. All participants gave written informed consent. The study conformed to the principles of the Declaration of Helsinki as reflected by an *a priori* approval of the Ethics Committee of the University of Greifswald. Of the 4,308 participants, 356 subjects with missing data of L-arginine, ADMA, or SDMA as well as with missing data for selected confounding factors were excluded from analyses. The final study population for the present analyses consisted of 3,952 subjects (1,936 men and 2,016 women). SHIP data are publically available for scientific and quality control purposes. Data usage can be applied for via www.community-medicine.de.

### Measurements

Information on age, sex, socio-demographic characteristics, and medical histories were assessed by computer-assisted personal interviews. Smoking status was assessed by self-report and categorized into current, former, and never-smokers. Participants who participated in physical exercise during summer or winter less than one hour a week were classified as being physically inactive. Waist circumference was measured to the nearest 0.1 cm using an inelastic tape midway between the lower rib margin and the iliac crest in the horizontal plane, with the subject standing comfortably with weight distributed evenly on both feet. The definition of diabetes mellitus was based on the self-reported use of antidiabetic medication [anatomic, therapeutic, and chemical (ATC) code: A10] in the last 7 days or a glycosylated haemoglobin level >6.5%. CVD definition was based on a self-reported history of angina pectoris, peripheral artery disease, stroke, or myocardial infarction. The definition of known liver disease was based on self-reported liver cirrhosis or atrophy of the liver. Additionally, all subjects with serum gamma-glutamyl-transferase (GGT), aspartate transaminase (AST), or alanine transaminase (ALT) levels > population mean +2*standard deviation were classified as subjects having liver disease. After a 5 minute resting period, systolic and diastolic blood pressure (BP) was measured three times on the right arm of seated subjects using a digital BP monitor (HEM-705CP, Omron Corporation, Tokyo, Japan) with each reading being followed by a further resting period of 3 minutes. The mean of the second and third measurement was used for analyses.

### Assays

Non-fasting blood samples were drawn from the cubital vein in the supine position. The samples were analyzed immediately or stored at −80°C. In addition, internal quality controls were performed at least daily. Total and high-density lipoprotein (HDL) cholesterol were measured photometrically (Hitachi 704; Roche, Mannheim, Germany). Low-density lipoprotein (LDL) cholesterol was measured by applying a precipitation procedure using dextran sulphate (Immuno, Heidelberg, Germany) on an Epos 5060 (Eppendorf, Hamburg, Germany). Triglycerides were determined enzymatically using reagents from Roche Diagnostics (Hitachi 717, Roche Diagnostics, Mannheim, Germany). Hypercholesterolemia were defined as LDL cholesterol ≥4.1 mmol/l or the self-reported use of statins (ATC code: C10AA). Serum creatinine levels were determined with the Jaffé method (Hitachi 717, Roche Diagnostics, Germany). The estimated glomerular filtration rate (eGFR) was calculated using the four-variable Modification of Diet in Renal Disease (MDRD) study equation: eGFR  = 186.3 * serum creatinine^−1.154^ * age^−0.203^ * (0.742 if female) [14], [15]. Serum AST, ALT, and GGT levels were measured photometrically (Hitachi 717, Roche Diagnostics GmbH, Mannheim, Germany). All assays were performed according to the manufacturers' recommendations by skilled technical personnel. Guanidino compounds, i.e. L-arginine, ADMA, and SDMA, were determined by liquid chromatography-tandem mass spectrometry (LC-MS/MS) by previously validated methods [16], [17]. In brief, 25 µL of serum were diluted with stable isotope labeled L-arginine and ADMA. Subsequently, proteins were precipitated with methanol and guanidino compounds were converted to their butyl esters. Concentrations of guanidino compounds were calculated with calibration curves (four levels, triplicates) and platewise quality controls (QC) were run (two levels, duplicates). Samples were re-analyzed for CVs and bias of QCs ≥15%. In every 10-years age-group, L-arginine, ADMA, or SDMA levels were categorized into three groups (low, intermediate, high) according to the sex-specific 33^th^ and 66^th^ percentiles.

### Follow-up of vital status

Information on vital status was collected from population registries at annual intervals from time of enrollment into the study through December 15, 2009. Subjects were censored at either death or failure to follow-up. The number of months between baseline examination and censoring was used as follow-up length. The median duration of follow-up was 10.1 years (25^th^ 9.3; 75^th^ 10.8). Death certificates were requested from the local health authority of the residence of death, and coded by a certified nosologist according to the International Classification of Diseases, 10^th^ revision (ICD10). Additionally, two internists (H.W. & M.D.) independently validated the underlying cause of death, and performed a joint reading together with a third internist (H.V.) in cases of disagreement. Cardiovascular disease (CVD) included codes I10 to I79, and cancer C00 to C97.

### Statistical Methods

Categorical data were expressed as percentages; continuous data were expressed as median (25^th^; 75^th^ quartile). Univariate analysis was performed with χ^2^ testing for categorical variables and Mann-Whitney-U-test for continuous distributions. Survival curves were estimated by the Kaplan-Meier method and compared using log-rank test. Multivariable Cox proportional hazard regression models with age as timescale were run to assess the associations between L-arginine, ADMA, or SDMA levels and all-cause as well as CV mortality. For CV mortality analyses, participants who died from other causes than the specific cause of interest were censored at the age at death. The models were adjusted for sex, physical activity, smoking, and waist circumference. Further confounders including diabetes, liver disease, eGFR, and systolic blood pressure were tested. Sensitivity analyses were run after the exclusion of six subjects who died within the first six months of follow-up, to account for acute disease. Finally, analyses were rerun and only in subjects ≥50 years. To check for non-linear relations, survivor functions were calculated by Cox regression using restricted cubic splines with 3 knots. Three knots were pre-specified located at the 5^th^, 50^th^, and 95^th^ percentile as recommended by Stone and Koo [18]. The model assumption for the Cox proportional hazards regression model was checked with Schoenfeld residuals and log of the negative log of survival plots. Hazard ratios (HR) with 95% confidence intervals (CI) were calculated. C-statistics for Cox models were calculated as previously described and the bias-corrected accelerated bootstrap resampling procedure of Efron and Tibshirani was used to obtain 95% confidence intervals [19]. A value of p<0.05 was considered statistically significant. Statistical analyses were performed with SAS 9.1 (SAS Institute Inc., Cary, NC, USA).

## Results

### General characteristics

Serum L-arginine derivatives and phenotypes were available from 3,952 participants of the SHIP cohort. 51% of the cohort represented females, characterized by a lower frequency of prevalent CVD as well as lower ADMA and SDMA serum concentrations (**Table 1**). ADMA as well as SDMA serum concentrations in participants with prevalent CVD (n = 442) were higher as compared with participants without CVD at baseline (n = 3,510), i.e. 0.70 (0.60; 0.79) µmol/L vs. 0.67 (0.59; 0.76) µmol/L ADMA and 0.48 (0.41; 0.58) µmol/L vs. 0.45 (0.39; 0.52) µmol/L SDMA (p<0.01 for both). No difference was observed for L-arginine (150 (119; 191) µmol/L vs. 152 (120; 188) µmol/L, p = 0.98). In unadjusted correlation analyses, L-arginine was correlated with blood cholesterol and ADMA (rho>0.1; p<0.001 for both, **Table 2**). ADMA showed the strongest correlations with SDMA, age, and eGFR followed by BMI and systolic blood pressure. For SDMA, in addition to a modest correlation with heart rate, similar correlations were found; effect sizes for the correlations with age, eGFR, and systolic blood pressure were more profound as compared with ADMA (**Table 2**).

**Table 1:** General characteristics of the study population stratified by sex.

	All subjects (n = 3,952)	Men (n = 1,936)	Women (n = 2,016)	p
Age, years	51 (37; 64)	52 (38; 65)	49 (36; 62)	<0.01
Smoking, %				<0.01
Never smoker	36.1	21.0	50.6	
Former smoker	33.8	45.5	22.5	
Current smoker	30.1	33.5	26.9	
Physical activity, %	42.4	41.2	43.6	0.14
Waist circumference, cm	89 (79; 99)	95 (88; 103)	82 (73; 92)	<0.01
Body-mass-index, kg/m^2^	26.9 (23.8; 30.1)	27.4 (24.9; 29.9)	26.2 (22.9; 30.2)	<0.01
Body-mass-index, %				
<25 kg/m^2^	34.2	26.1	41.8	
25–30 kg/m^2^	40.5	49.4	32.0	
≥30 kg/m^2^	25.3	24.5	26.1	
Blood pressure, mmHg				
Systolic	135 (121; 149)	141 (130; 153)	127 (115; 143)	<0.01
Diastolic	83 (76; 91)	85 (78; 93)	80 (74; 88)	<0.01
eGFR, ml/min/1.73 m^2^	79 (70; 89)	83 (74; 93)	75 (67; 84)	<0.01
eGFR, %				
<30 ml/min/1.73 m^2^	0.3	0.3	0.2	
30–60 ml/min/1.73 m^2^	8.7	6.3	11.1	
60–100 ml/min/1.73 m^2^	83.6	81.8	85.2	
>100 ml/min/1.73 m^2^	7.4	11.5	3.6	
Blood lipids, [mmol/l]				
Total cholesterol	5.69 (4.93; 6.48)	5.70 (4.95; 6.44)	5.67 (4.90; 6.50)	0.80
HDL cholesterol	1.39 (1.15; 1.70)	1.25 (1.05; 1.50)	1.54 (1.28; 1.84)	<0.01
LDL cholesterol	3.48 (2.76; 4.26)	3.57 (2.85; 4.27)	3.43 (2.69; 4.22)	<0.01
Triglycerides	1.48 (1.02; 2.26)	1.69 (1.16; 2.60)	1.32 (0.93; 1.91)	<0.01
Prevalent disease, %				
CVD	11.2	12.2	10.2	0.05
Hypertension	52.0	62.5	41.8	<0.01
Hypercholesterolemia	34.7	37.4	32.0	<0.01
Diabetes	9.8	11.4	8.3	<0.01
Liver disease	5.6	5.7	5.5	0.84
Arginine derivatives				
L-Arginine, µmol/l	152 (120; 188)	151 (119; 185)	153 (121; 190)	0.11
ADMA, µmol/l	0.67 (0.59; 0.76)	0.68 (0.60; 0.77)	0.67 (0.59; 0.76)	0.01
SDMA, µmol/l	0.45 (0.39; 0.53)	0.47 (0.40; 0.54)	0.44 (0.38; 0.51)	<0.01

Continuous data are given as median (25th; 75th quartile); nominal data are given as percentages. χ^2^-test (nominal data) or Kruskal-Wallis test (interval data) were used. eGFR  =  estimated glomerular filtration rate; HDL  =  high density lipoprotein; CVD  =  cardiovascular disease; CKD  =  chronic kidney disease; ADMA  =  asymmetric dimethylarginine; SDMA  =  symmetric dimethylarginine.

**Table 2:** Cross-sectional analyses between phenotypes and arginine derivatives.

	L-Arginine	ADMA	SDMA
Age, years	−0.025 *(0.14)*	0.246 *(<.001)*	0.308 *(<.001)*
BMI, m^2^/kg	0.06 (*<.001)*	0.132 *(<.001)*	0.07 *(<.001)*
Blood pressure			
Systolic	0.039 *(0.02)*	0.126 *(<.001)*	0.149 *(<.001)*
Diastolic	0.066 *(<.001)*	0.072 *(<.001)*	0.042 (*0.01)*
Heart rate	0.036 (*0.04*)	−0.039 *(0.01)*	−0.105 (*<.001)*
Blood lipids			
Cholesterol	0.101 (*<.001)*	0.080 *(<.001)*	0.072 (*<.001*)
HDL	0.008 (*0.65*)	−0.070 (*<.001*)	−0.063 *(<.001)*
Triglycerides	0.026 (*0.13*)	0.060 (*<.001*)	0.050 (*<.01*)
Glucose	0.020 (*0.25*)	0.083 (*<.001*)	0.039 (*0.01*)
hs-CRP	0.084 (*<.001*)	0.042 *(0.04)*	0.025 *(0.12)*
eGFR, ml/min/1.73 m^2^	0.009 (*0.60*)	−0.179 (*<.001*)	−0.372 (*<.001*)
Arginine derivatives			
L-Arginine, µmol/L		0.114 (*<.001*)	0.054 (*<.01*)
ADMA, µmol/l	0.114 (*<.001*)		0.524 (*<.001*)

Data presented as rho- and (*p-values*). BMI  =  body mass index; HDL  =  high density lipoprotein; hsCRP  =  high sensitive C reactive protein; eGFR  =  estimated glomerular filtration rate; ADMA  =  asymmetric dimethylarginine; SDMA  =  symmetric dimethylarginine.

### Association of L-arginine derivatives with all-cause mortality

**Figure 1:**
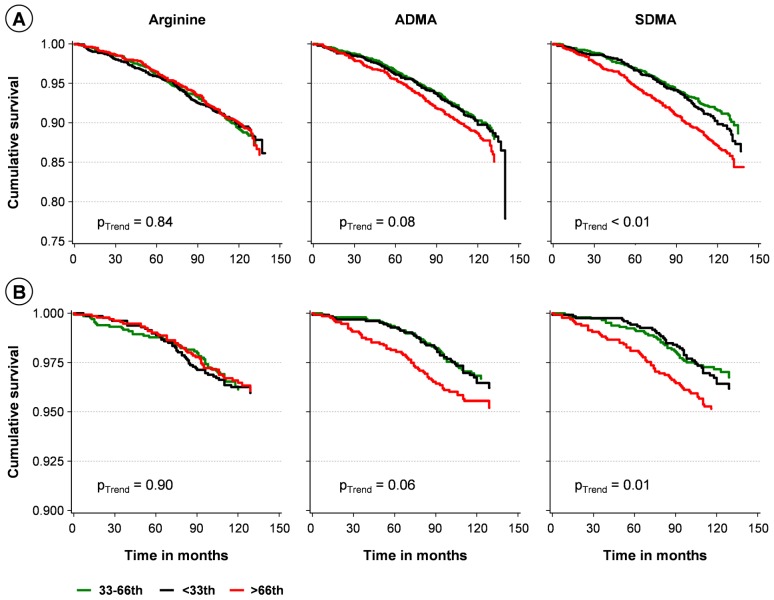
Survival curves for all-cause (A) and CV (B) mortality by levels of L-arginine, asymmetric dimethylarginine (ADMA) and symmetric dimethylarginine (SDMA). L-Arginine and L-arginine derivates levels were categorized into three levels according to the age- and sex-specific 33^th^ and 66^th^ percentile. Log-rank tests for trend were performed.

**Figure 2:**
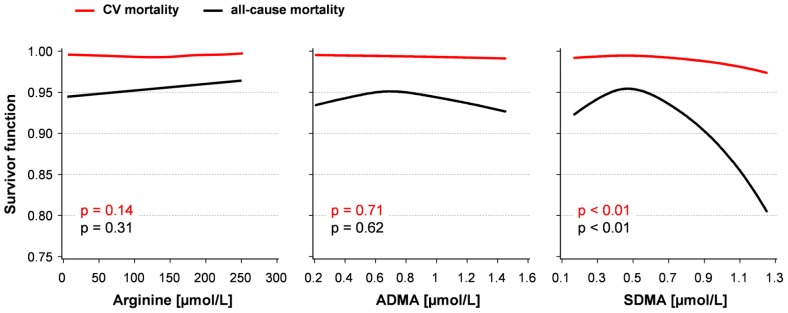
Survivor function depending on levels of L-arginine, asymmetric dimethylarginine (ADMA) and symmetric dimethylarginine (SDMA) calculated by Cox regression adjusted for sex, physical activity, smoking, and waist circumference. Age was used as timescale. Curves represent the 10 year survival of a 50 year old men with the following conditions: waist circumference  = 95 cm, former smoker and no physical activity.

**Figure 3:**
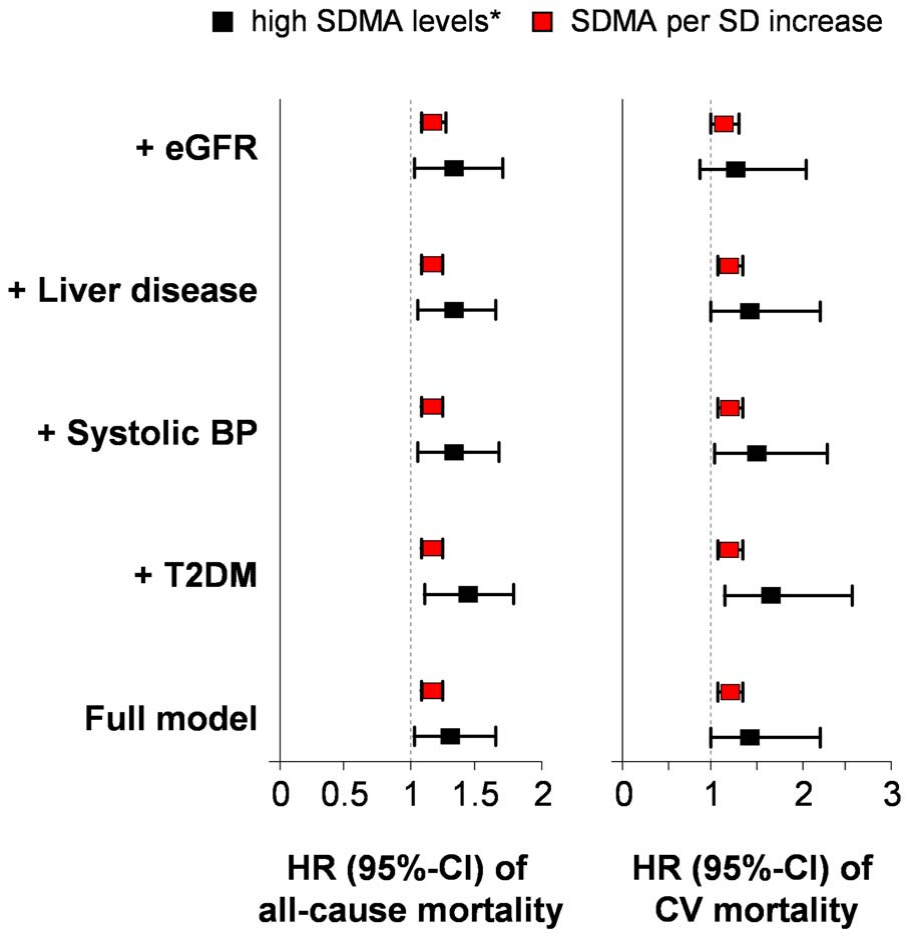
Hazard ratio (HR) with 95% CI (confidence interval) of all-cause and cardiovascular (CV) mortality by levels of symmetric dimethylarginine (SDMA). The full model was adjusted for sex, physical activity, smoking, and waist circumference. Age was used as timescale. To the full model the following covariates were added separately: diabetes (T2DM), systolic blood pressure (BP), liver disease, and estimated glomerular filtration rate (eGFR). High SDMA levels were categorized according to the age- and sex-specific 66^th^ percentile (reference: low: <33^th^ percentile).

Kaplan-Meier analyses using categorized levels demonstrated that all-cause mortality was higher in subjects with high SDMA levels compared to subjects with low or intermediate levels (**Figure 1** **A**). No significant mortality differences were found across L-arginine and ADMA categories, even though a trend for ADMA was observed (p = 0.08). The median SDMA serum concentration of subjects who died during follow-up was higher as compared with those who survived, 0.52 (0.43; 0.62) µmol/L vs. 0.45 (0.38; 0.52) µmol/L (p<0.01). In sex-adjusted as well as multivariable Cox proportional hazard models (further adjustment for physical activity, smoking and waist circumference) SDMA was associated with all-cause mortality (**Table 3**). SDMA levels of the highest tertile, i.e. 0.56 (0.51; 0.63) µmol/L (**Table S1**) were related to a 66% higher all-cause mortality risk compared to intermediate levels. Also continuous analyses revealed a slightly inverse J-shaped association between SDMA levels and mortality risk with a strong decline in higher range of SDMA (**Figure 2**). An increase of one SD in SDMA was associated with a 16% higher mortality risk (**Table 3**). Similar results were obtained after additional inclusion of confounding factors, i.e. diabetes, liver disease, eGFR as marker of renal function, and systolic blood pressure (**Figure 3**, **Table S2**). The association of SDMA with all-cause mortality was stratified by median eGFR (79 (70; 89) ml/min/1.73 m^2^). Mortality risk was in particular increased in the highest tertile of SDMA for participants with eGFR below the median (**Figure S1, Table S4**). C-statistics for Cox models including conventional CV risk factors showed an area under the curve (AUC) of 0.85 and 0.86 without and with inclusion of SDMA, respectively (p>0.05).

**Table 3:** Association of L-arginine and L-arginine derivates with all-cause or cause-specific mortality.

	All-cause mortality				CV mortality			
	sex-adjusted		adjusted†		sex-adjusted		adjusted†	
	HR (95%-CI)	p	HR (95%-CI)	p	HR (95%-CI)	p	HR (95%-CI)	p
L-Arginine*								
per SD increase	0.94 (0.85; 1.04)	0.24	0.92 (0.83; 1.02)	0.13	0.88 (0.73; 1.05)	0.17	0.87 (0.72; 1.04)	0.12
Arginine (ref.: <33^th^)								
33–66^th^	1.01 (0.80; 1.27)	0.95	0.96 (0.76; 1.21)	0.72	0.95 (0.63; 1.43)	0.80	0.91 (0.60; 1.36)	0.64
>66^th^	0.94 (0.74; 1.19)	0.61	0.89 (0.70; 1.12)	0.31	0.84 (0.56; 1.26)	0.39	0.78 (0.52; 1.18)	0.24
**ADMA**								
per SD increase	1.05 (0.95; 1.15)	0.34	1.02 (0.93; 1.12)	0.72	1.09 (0.93; 1.28)	0.29	1.07 (0.91; 1.26)	0.41
ADMA (ref.: <33^th^)								
33–66^th^	0.94 (0.74; 1.19)	0.60	0.93 (0.73; 1.18)	0.56	0.91 (0.59; 1.40)	0.67	0.91 (0.59; 1.40)	0.67
>66^th^	1.19 (0.94; 1.50)	0.15	1.10 (0.87; 1.39)	0.41	1.37 (0.92; 2.06)	0.12	1.30 (0.86; 1.94)	0.21
**SDMA**								
per SD increase	1.15 (1.07; 1.25)	<0.01	1.16 (1.07; 1.25)	<0.01	1.20 (1.06; 1.35)	<0.01	1.19 (1.05; 1.35)	0.01
SDMA (ref.: 33–66^th^)								
<33^th^	1.21 (0.95; 1.55)	0.12	1.26 (0.99; 1.62)	0.06	1.16 (0.75; 1.79)	0.50	1.19 (0.77; 1.84)	0.44
>66^th^	1.62 (1.28; 2.03)	<0.01	1.66 (1.32; 2.09)	<0.01	1.70 (1.14; 2.54)	<0.01	1.76 (1.18; 2.63)	<0.01

HR  =  hazard ratio; CI  =  confidence interval. ADMA  =  asymmetric dimethylarginine; SDMA  =  symmetric dimethylarginine. L-Arginine and L-arginine derivates levels were categorized into three levels according to the age- and sex-specific 33th and 66th percentile (for more detail see supplement). * Subjects with L-arginine levels upper limit of quantification were excluded.

† Model was adjusted for sex, physical activity, smoking and waist circumference. Age was used as timescale.

### Association of L-arginine derivatives with cardiovascular mortality

Supporting the findings for all-cause mortality, Kaplan-Meier analyses showed that subjects with high SDMA levels had a higher incidence of CV mortality than subjects with low or intermediate levels (**Figure 1B**). Adjusted Cox regression analyses substantiated this association; an increase of SDMA levels of one SD was related to a 19% increase in CV mortality risk (**Table 3**). The additional inclusion of confounding factors did not change the direction of the association (**Figure 3**, **Table S3**). C-statistics for Cox models including conventional CV risk factors showed similar AUCs without and with inclusion of SDMA. Neither L-arginine nor ADMA was related to CV mortality (**Table 3**). Sensitivity analyses were run after the exclusion of subjects who died within the first six months of follow-up (n = 6) and subjects <50 years (n = 2,019 with 399 deaths). The reported associations of SDMA with all-cause mortality as well as CV mortality were confirmed, and the estimates did not substantially change. The same set of analyses was run for cancer mortality, but no associations for L-arginine, SDMA, or ADMA were found

## Discussion

The main findings of our investigation are: (i) In cross-sectional analyses, SDMA is not only related to age and renal function, but also to systolic blood pressure and heart rate. (ii) In longitudinal analyses, SDMA was found to be an independent predictor of all-cause and CV mortality. This observation was still evident after adjustment for diabetes, liver disease, or renal function.

For decades, SDMA has been considered as the biological inactive congener of ADMA [20]. Both dimethylarginines are formed by methylation of protein residues; ADMA as a product of PRMT type I and SDMA of PRMT type II. ADMA is a competitive inhibitor of NOS and cationic amino acid uptake thereby reducing NO production from NOS, whereas SDMA only indirectly influences NO bioavailability by reducing substrate availability due to inhibition of L-arginine uptake [6], [7]. SDMA has been shown to activate stored-opened Ca^2+^-channeles in monocytes, which might contribute to monocyte activation [21]. Other biological functions of SDMA are still unknown. To date, epidemiological data on both ADMA and SDMA is sparse. Previously, an association of ADMA with all-cause mortality was reported in the Framingham Heart Study, however, such an association was not evident for CV mortality [9]. In the population-based Bruneck Study a high risk for a composite CV endpoint including non-fatal stroke, transient ischemic attack, and myocardial infarction as well as CV death, was reported for individuals with SDMA in the highest percentile (>0.8 µmol/l) [22]. In line with this, recent results from the multi ethnic cohort of the Dallas Heart Study related high levels of SDMA to CV and all-cause mortality [10]. Of note, the Dallas Heart Study comprises approximately 50% Afro-Americans, with an over-proportional number of CV and all-cause deaths in this sub-group. Interestingly, in the present investigation of the population of West Pomerania in Germany, SDMA was also associated with all-cause and CV mortality. The observation, that SHIP participants with high SDMA have an increased risk of CV death, is most likely attributed to underlying CVD. Supporting this hypothesis, recent findings from the LURIC (Ludwigshafen Risk and Cardiovascular Health) Study independently associated SDMA with increased CV and all-cause mortality in patients undergoing coronary angiography [23]. Siegerink et al, reported a higher incidence of recurrent myocardial infarction or stroke as well as a higher mortality rate in patients with increased plasma SDMA [24]. Moreover, recent results from the Dallas Heart Study related high SDMA to a higher prevalence of CV risk factors and for the first time to subclinical markers of atherosclerosis, i.e., coronary artery calcification, and abdominal aortic wall thickness [10]. In our cross-sectional analyses in the SHIP population we noticed a correlation between SDMA and systolic BP as well as heart rate (**Table 2**). Recently, genome-wide association studies revealed a novel link between SDMA plasma concentrations and single nucleotide polymorphisms (SNPs) in the *AGXT2* gene. Tellingly, these SNPs in the *AGXT2* gene were associated with heart rate variability [25], providing evidence for a link between SDMA, AGXT2, and sympathetic activity.

SDMA is strongly related to renal function (reviewed in [26]). SDMA is eliminated by the kidney and its level increases already at early stages of renal impairment due to a reduced blood clearance [27]. Therefore, SDMA has been suggested as a highly sensitive marker of renal (dys)function, which is independent of muscle mass or body surface [28]. In our study population, adjustment of the association between SDMA and mortality for renal function did not weaken the observation (**Table S2**). This implies that SDMA predicts mortality independently of eGFR in our study cohort. However, mortality risk was in particular increased in participants with eGFR below the median (**Figure S1, Table S4**).

A limitation of our study is the younger age of the SHIP population investigated as compared to the age of participants of previously investigated offspring-cohort from the Framingham Heart Study (i.e., 51 vs. 59 years, respectively) [9]. This might explain, why we did not observe a relation of ADMA to CV outcome nor to death in the present study population. The strengths of the present study are the population-based approach, the accurate assessment of causes of death based on ICD-10 coding, and the detailed assessment of metabolic and CV risk factors. In a large cohort, we identified high SDMA as independently associated with CV and all-cause mortality; however, despite adjustment, unidentified confounding effects cannot be ruled out, and SDMA did not improve mortality risk prediction incrementally over conventional risk factors. Therefore, additional epidemiological, clinical, and experimental studies are required for validation of our results and investigation of underlying (patho)mechanisms.

### Conclusion

Our data provide new insights into a prognostic as well as mechanistic link between SDMA and CV outcome in a large population-based cohort. Replication of the present findings in other cohorts as well as pathomechanistic investigations using mouse models of impaired or increased SDMA metabolism are needed to further elucidate the biological importance of SDMA.

## Supporting Information

Figure S1 **Survival curves for all-cause mortality by levels of symmetric dimethylarginine (SDMA) for subjects with estimated glomerular filtration rate (eGFR) < median or ≥ median (79 ml/min/1.73 m2).** SDMA levels were categorized into three levels according to the age- and sex-specific 33th and 66th percentile. Log-rank tests for trend were performed.
(TIF)

Table S1 **General characteristics of the study population by symmetric dimethylarginine (SDMA) levels.**
(DOC)

Table S2 **Hazard ratios (HR) of L-arginine and arginine derivate levels for all-cause mortality adjusted for additional confounders.**
(DOC)

Table S3 **Hazard ratios (HR) of L-arginine and arginine derivate levels for CV mortality adjusted for additional confounders.**
(DOC)

Table S4 **Hazard ratios (HR) of SDMA levels for all-cause stratified by median GFR.**
(DOC)
