# Supplementary figures and images for: Incidence of All-Cause and Cardiovascular Mortality Predicted by Symmetric Dimethylarginine in the Population-Based Study of Health in Pomerania
Source: PLoS One. 2014 May 12;9(5):e96875. doi: 10.1371/journal.pone.0096875 (PMC4018357; doi:10.1371/journal.pone.0096875)

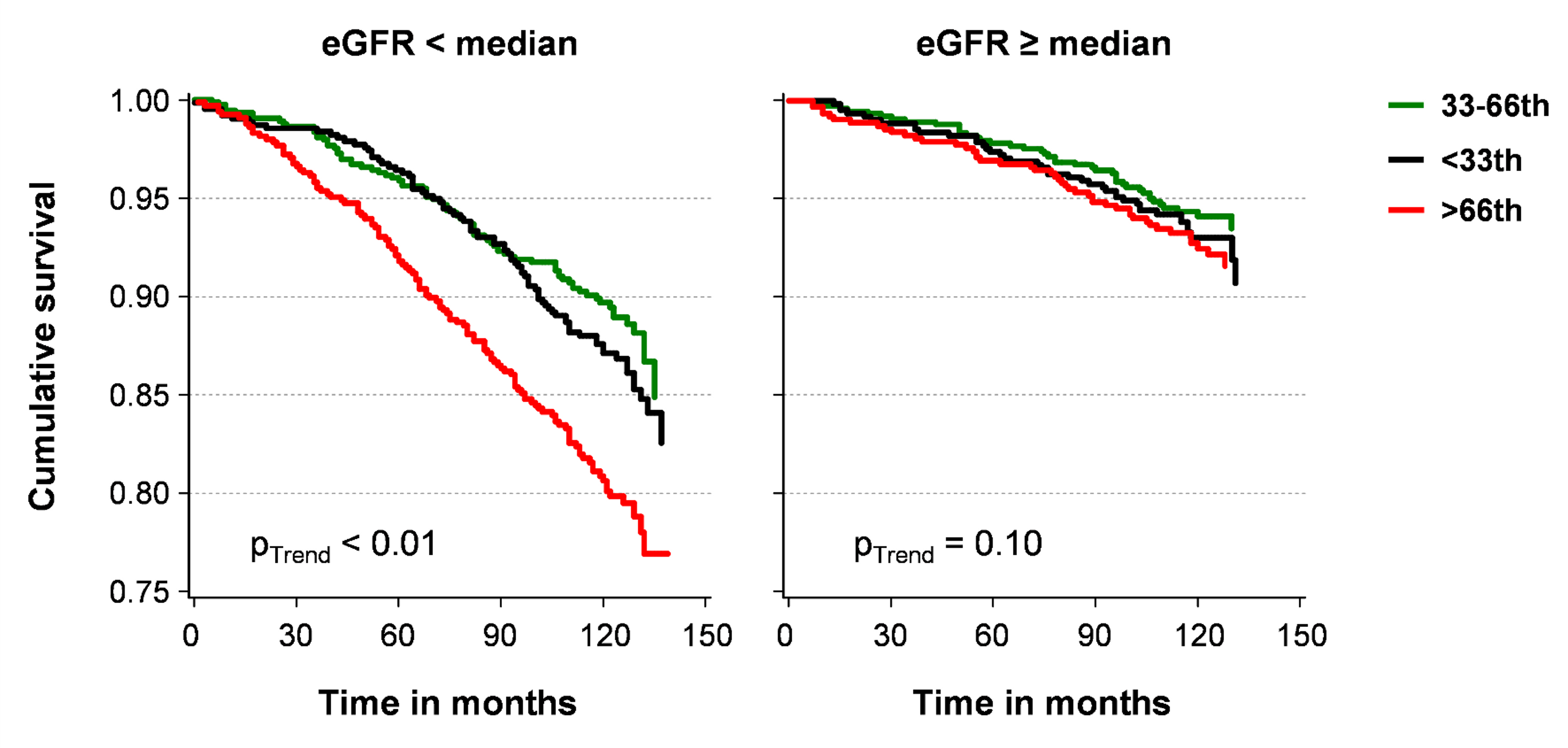

Supplement: Figure S1 — Survival curves for all-cause mortality by levels of symmetric dimethylarginine (SDMA) for subjects with estimated glomerular filtration rate (eGFR) < median or ≥ median (79 ml/min/1.73 m2). SDMA levels were categorized into three levels according to the age- and sex-specific 33th and 66th percentile. Log-rank tests for trend were performed. (TIF) [file pone.0096875.s001.tif]
