# Supplementary material for: Incidence of All-Cause and Cardiovascular Mortality Predicted by Symmetric Dimethylarginine in the Population-Based Study of Health in Pomerania
Source: PLoS One. 2014 May 12;9(5):e96875. doi: 10.1371/journal.pone.0096875 (PMC4018357; doi:10.1371/journal.pone.0096875)
Supplement: Table S1 — General characteristics of the study population by symmetric dimethylarginine (SDMA) levels. (DOC) [file pone.0096875.s002.doc]

**Table S1. General characteristics of the study population by symmetric dimethylarginine (SDMA) levels.**

|  | **SDMA levels** | | | |
| --- | --- | --- | --- | --- |
|  | **<33^th^ percentile (n = 1223)** | **33-66^th^ percentile (n = 1450)** | **>66^th^ percentile (n = 1208)** | **p** |
| Age, years | 50 (36; 63) | 51 (37; 64) | 50 (36; 64) | 0.45 |
| Smoking, % |  |  |  | 0.91 |
| Never smoker | 36.5 | 36.1 | 34.4 |  |
| Former smoker | 34.0 | 33.0 | 35.7 |  |
| Current smoker | 29.5 | 30.9 | 29.9 |  |
| Physical inactive, % | 42.2 | 42.6 | 42.4 | 0.97 |
| Waist circumference, cm | 90 (80; 100) | 90 (79; 99) | 88 (78; 98)* | 0.03 |
| Diastolic BP, mmHG | 83 (76; 91) | 82 (75; 90) | 83 (75; 91) | 0.33 |
| Systolic BP, mmHG | 136 (122; 150) | 135 (121; 149) | 135 (120; 149) | 0.13 |
| eGFR, ml/min/1.73m^2^ | 82 (73; 92)* | 80 (70; 89) | 75 (65; 84)* | <0.01 |
| Diabetes, % | 13.4* | 8.3 | 8.0 | <0.01 |
| Liver disease, % | 6.0 | 5.3 | 5.6 | 0.76 |
| L-Arginine, µmol/l^†^ | 145 (113; 184)* | 154 (124; 187) | 157 (123; 192) | <0.01 |
| ADMA, µmol/l | 0.60 (0.53; 0.69)* | 0.67 (0.60; 0.75) | 0.75 (0.66; 0.84)* | <0.01 |
| SDMA, µmol/l | 0.36 (0.32; 0.39) | 0.45 (0.42; 0.48) | 0.56 (0.51; 0.63) | <0.01 |

Continuous data are given as median (25th; 75th quartile); nominal data are given as percentages.
χ^2^-test (nominal data) or Kruskal-Wallis test (interval data) were used. eGFR = estimated glomerular filtration rate; ADMA = asymmetric dimethylarginine. SDMA levels were categorized into three levels according to the age- and sex-specific 33th and 66th percentile (for more details see supplement). † Subjects with arginine levels upper limit of quantification were excluded. *p <0.05 for comparison against subjects with intermediate SDMA levels.
