# Supplementary material for: Incidence of All-Cause and Cardiovascular Mortality Predicted by Symmetric Dimethylarginine in the Population-Based Study of Health in Pomerania
Source: PLoS One. 2014 May 12;9(5):e96875. doi: 10.1371/journal.pone.0096875 (PMC4018357; doi:10.1371/journal.pone.0096875)
Supplement: Table S2 — Hazard ratios (HR) of L-arginine and arginine derivate levels for all-cause mortality adjusted for additional confounders. (DOC) [file pone.0096875.s003.doc]

**Table S2**. Hazard ratios (HR) of L-arginine and arginine derivate levels for all-cause mortality adjusted for additional confounders.

|  | **Hazard ratio (95%-CI) for all-cause mortality** | | | | | | | | | | | | | |
| --- | --- | --- | --- | --- | --- | --- | --- | --- | --- | --- | --- | --- | --- | --- |
|  | **#** | |  | **# + Diabetes** | |  | **# + Liver disease** | |  | **# + GFR** | |  | **# + systolic BP** | |
|  | **HR (95%-CI)** | **p** |  | **HR (95%-CI)** | **p** |  | **HR (95%-CI)** | **p** |  | **HR (95%-CI)** | **p** |  | **HR (95%-CI)** | **p** |
| **L-Arginine** |  |  |  |  |  |  |  |  |  |  |  |  |  |  |
| per SD increase* | 0.92 (0.83; 1.02) | 0.13 |  | 0.92 (0.83; 1.02) | 0.12 |  | 0.92 (0.83; 1.03) | 0.14 |  | 0.92 (0.83; 1.03) | 0.14 |  | 0.92 (0.82; 1.02) | 0.10 |
| Arginine (ref.: <33th) | |  |  |  |  |  |  |  |  |  |  |  |  |  |
| 33-66th | 0.96 (0.76; 1.21) | 0.72 |  | 0.99 (0.78; 1.25) | 0.91 |  | 0.96 (0.76; 1.21) | 0.73 |  | 0.96 (0.76; 1.21) | 0.71 |  | 0.95 (0.75; 1.21) | 0.70 |
| >66th | 0.89 (0.70; 1.12) | 0.31 |  | 0.89 (0.70; 1.12) | 0.32 |  | 0.89 (0.70; 1.12) | 0.32 |  | 0.88 (0.70; 1.12) | 0.30 |  | 0.88 (0.69; 1.11) | 0.28 |
|  |  |  |  |  |  |  |  |  |  |  |  |  |  |  |
| **ADMA** |  |  |  |  |  |  |  |  |  |  |  |  |  |  |
| per SD increase | 1.02 (0.93; 1.12) | 0.72 |  | 1.03 (0.94; 1.13) | 0.57 |  | 1.02 (0.93; 1.12) | 0.72 |  | 1.01 (0.92; 1.11) | 0.80 |  | 1.02 (0.93; 1.12) | 0.71 |
| ADMA (ref.: <33th) | |  |  |  |  |  |  |  |  |  |  |  |  |  |
| 33-66th | 0.93 (0.73; 1.18) | 0.56 |  | 0.97 (0.76; 1.23) | 0.80 |  | 0.95 (0.75; 1.21) | 0.68 |  | 0.93 (0.73; 1.18) | 0.55 |  | 0.93 (0.74; 1.18) | 0.57 |
| >66th | 1.10 (0.87; 1.39) | 0.41 |  | 1.13 (0.89; 1.42) | 0.32 |  | 1.11 (0.88; 1.40) | 0.40 |  | 1.09 (0.86; 1.38) | 0.46 |  | 1.10 (0.87; 1.39) | 0.41 |
|  |  |  |  |  |  |  |  |  |  |  |  |  |  |  |
| **SDMA** |  |  |  |  |  |  |  |  |  |  |  |  |  |  |
| per SD increase | 1.16 (1.07; 1.25) | <0.01 |  | 1.16 (1.08; 1.25) | <0.01 |  | 1.16 (1.07; 1.25) | <0.01 |  | 1.17 (1.07; 1.28) | <0.01 |  | 1.16 (1.07; 1.25) | <0.01 |
| SDMA (ref.: 33-66th) | |  |  |  |  |  |  |  |  |  |  |  |  |  |
| <33th | 1.26 (0.99; 1.62) | 0.06 |  | 1.18 (0.92; 1.52) | 0.18 |  | 1.24 (0.97; 1.58) | 0.09 |  | 1.26 (0.99; 1.62) | 0.07 |  | 1.26 (0.98; 1.61) | 0.07 |
| >66th | 1.66 (1.32; 2.09) | <0.01 |  | 1.68 (1.33; 2.11) | <0.01 |  | 1.64 (1.30; 2.06) | <0.01 |  | 1.67 (1.31; 2.12) | <0.01 |  | 1.67 (1.32; 2.10) | <0.01 |

HR = hazard ratio; CI = confidence interval. ADMA = asymmetric dimethylarginine; SDMA = symmetric dimethylarginine. L-Arginine and arginine derivate levels were categorized into three levels according to the age- and sex-specific 33th and 66th percentile. *Subjects with arginine levels upper limit of quantification were excluded. # Model was adjusted for sex, physical activity, smoking and waist circumference. Age was used as timescale. Covariates were added separately to the model.
