# Supplementary material for: Incidence of All-Cause and Cardiovascular Mortality Predicted by Symmetric Dimethylarginine in the Population-Based Study of Health in Pomerania
Source: PLoS One. 2014 May 12;9(5):e96875. doi: 10.1371/journal.pone.0096875 (PMC4018357; doi:10.1371/journal.pone.0096875)
Supplement: Table S3 — Hazard ratios (HR) of L-arginine and arginine derivate levels for CV mortality adjusted for additional confounders. (DOC) [file pone.0096875.s004.doc]

**Table S3**. Hazard ratios (HR) of L-arginine and arginine derivate levels for CV mortality adjusted for additional confounders.

|  | **Hazard ratio (95%-CI) for CV mortality** | | | | | | | | | | | | | |
| --- | --- | --- | --- | --- | --- | --- | --- | --- | --- | --- | --- | --- | --- | --- |
|  | **#** | |  | **# + Diabetes** | |  | **# + Liver disease** | |  | **# + GFR** | |  | **# + systolic BP** | |
|  | **HR (95%-CI)** | **p** |  | **HR (95%-CI)** | **p** |  | **HR (95%-CI)** | **p** |  | **HR (95%-CI)** | **p** |  | **HR (95%-CI)** | **p** |
| **L-Arginine** |  |  |  |  |  |  |  |  |  |  |  |  |  |  |
| per SD increase* | 0.87 (0.72; 1.04) | 0.12 |  | 0.87 (0.73; 1.04) | 0.13 |  | 0.87 (0.73; 1.04) | 0.13 |  | 0.88 (0.73; 1.05) | 0.16 |  | 0.86 (0.71; 1.03) | 0.09 |
| Arginine (ref.: <33th) | |  |  |  |  |  |  |  |  |  |  |  |  |  |
| 33-66th | 0.91 (0.60; 1.36) | 0.64 |  | 0.96 (0.63; 1.44) | 0.83 |  | 0.91 (0.60; 1.37) | 0.65 |  | 0.91 (0.60; 1.37) | 0.64 |  | 0.90 (0.60; 1.35) | 0.61 |
| >66th | 0.78 (0.52; 1.18) | 0.24 |  | 0.79 (0.52; 1.19) | 0.26 |  | 0.78 (0.52; 1.18) | 0.23 |  | 0.79 (0.52; 1.19) | 0.25 |  | 0.77 (0.51; 1.16) | 0.20 |
|  |  |  |  |  |  |  |  |  |  |  |  |  |  |  |
| **ADMA** |  |  |  |  |  |  |  |  |  |  |  |  |  |  |
| per SD increase | 1.07 (0.91; 1.26) | 0.41 |  | 1.07 (0.92; 1.26) | 0.38 |  | 1.08 (0.91; 1.27) | 0.38 |  | 1.05 (0.89; 1.24) | 0.56 |  | 1.08 (0.91; 1.27) | 0.38 |
| ADMA (ref.: <33th) | |  |  |  |  |  |  |  |  |  |  |  |  |  |
| 33-66th | 0.91 (0.59; 1.40) | 0.67 |  | 0.98 (0.64; 1.51) | 0.92 |  | 0.95 (0.61; 1.46) | 0.80 |  | 0.90 (0.58; 1.38) | 0.62 |  | 0.91 (0.59; 1.41) | 0.68 |
| >66th | 1.30 (0.86; 1.94) | 0.21 |  | 1.31 (0.87; 1.97) | 0.19 |  | 1.32 (0.88; 1.98) | 0.18 |  | 1.25 (0.83; 1.88) | 0.29 |  | 1.30 (0.87; 1.95) | 0.20 |
|  |  |  |  |  |  |  |  |  |  |  |  |  |  |  |
| **SDMA** |  |  |  |  |  |  |  |  |  |  |  |  |  |  |
| per SD increase | 1.19 (1.05; 1.35) | 0.01 |  | 1.20 (1.07; 1.35) | <0.01 |  | 1.19 (1.06; 1.35) | <0.01 |  | 1.14 (0.99; 1.32) | 0.08 |  | 1.21 (1.06; 1.36) | <0.01 |
| SDMA (ref.: 33-66th) | |  |  |  |  |  |  |  |  |  |  |  |  |  |
| <33th | 1.19 (0.77; 1.84) | 0.44 |  | 1.06 (0.69; 1.65) | 0.78 |  | 1.17 (0.75; 1.81) | 0.49 |  | 1.23 (0.79; 1.91) | 0.36 |  | 1.18 (0.76; 1.83) | 0.46 |
| >66th | 1.76 (1.18; 2.63) | <0.01 |  | 1.81 (1.21; 2.71) | <0.01 |  | 1.73 (1.16; 2.58) | <0.01 |  | 1.62 (1.06; 2.46) | 0.03 |  | 1.79 (1.20; 2.67) | <0.01 |

HR = hazard ratio; CI = confidence interval. ADMA = asymmetric dimethylarginine; SDMA = symmetric dimethylarginine. L-Arginine and arginine derivate levels were categorized into three levels according to the age- and sex-specific 33th and 66th percentile. * Subjects with arginine levels upper limit of quantification were excluded. # Model was adjusted for sex, physical activity, smoking and waist circumference. Age was used as timescale. Covariates were added separately to the model.
