# Supplementary material for: Incidence of All-Cause and Cardiovascular Mortality Predicted by Symmetric Dimethylarginine in the Population-Based Study of Health in Pomerania
Source: PLoS One. 2014 May 12;9(5):e96875. doi: 10.1371/journal.pone.0096875 (PMC4018357; doi:10.1371/journal.pone.0096875)
Supplement: Table S4 — Hazard ratios (HR) of SDMA levels for all-cause stratified by median GFR. (DOC) [file pone.0096875.s005.doc]

**Table S4**. Hazard ratios (HR) of SDMA levels for all-cause stratified by median GFR.

|  | **All-cause mortality** | | | | |
| --- | --- | --- | --- | --- | --- |
|  | **sex-adjusted** | |  | **adjusted^†^** | |
| **SDMA** | **HR (95%-CI)** | **p** |  | **HR (95%-CI)** | **p** |
| ***GFR ≥ Median (N = 1968; number of death = 132)*** | | | | |  |
| SDMA (ref.: 33-66^th^) | |  |  |  |  |
| <33^th^ | 1.30 (0.85; 1.99) | 0.23 |  | 1.33 (0.87; 2.04) | 0.18 |
| >66^th^ | 1.51 (0.99; 2.30) | 0.06 |  | 1.61 (1.05; 2.45) | 0.03 |
| ***GFR < Median (N = 1984; number of death = 294)*** | | | | |  |
| SDMA (ref.: 33-66^th^) | |  |  |  |  |
| <33^th^ | 1.16 (0.85; 1.57) | 0.35 |  | 1.21 (0.89; 1.65) | 0.22 |
| >66^th^ | 1.83 (1.38; 2.42) | <0.01 |  | 1.88 (1.43; 2.49) | <0.01 |

HR = hazard ratio; CI = confidence interval. SDMA = symmetric dimethylarginine. SDMA was categorized into three levels according to the age- and sex-specific 33th and 66th percentile.
**^†^** Model was adjusted for sex, physical activity, smoking and waist circumference. Age was used as timescale. Covariates were added separately to the model.
